# Development and validation of a clinic-radiological model to predict tumor spread through air spaces in stage I lung adenocarcinoma

**DOI:** 10.1186/s40644-024-00668-w

**Published:** 2024-02-09

**Authors:** Zhaisong Gao, Pingping An, Runze Li, Fengyu Wu, Yuhui Sun, Jie Wu, Guangjie Yang, Zhenguang Wang

**Affiliations:** 1 Department of Nuclear Medicine, Affiliated Hospital of Qingdao University, Qingdao, Shandong China; 2 Qingdao Medical College, Qingdao University, Qingdao, Shandong China; 3 Department of Thyroid Disease, Qingdao Municipal Hospital Group East Hospital, Qingdao Municipal Hospital Group, Qingdao, Shandong China; 4 Department of Thoracic Surgery, Affiliated Hospital of Qingdao University, Qingdao, Shandong China; 5 Department of Pathology, Affiliated Hospital of Qingdao University, Qingdao, Shandong China

**Keywords:** Lung, Adenocarcinoma, Positron emission tomography, Computed tomography, Invasion

## Abstract

**Objectives:**

Tumor spread through air spaces (STAS) is associated with poor prognosis and impacts surgical options. We aimed to develop a user-friendly model based on 2-[^18^F] FDG PET/CT to predict STAS in stage I lung adenocarcinoma (LAC).

**Materials and methods:**

A total of 466 stage I LAC patients who underwent 2-[^18^F] FDG PET/CT examination and resection surgery were retrospectively enrolled. They were split into a training cohort (*n* = 232, 20.3% STAS-positive), a validation cohort (*n* = 122, 27.0% STAS-positive), and a test cohort (*n* = 112, 29.5% STAS-positive) according to chronological order. Some commonly used clinical data, visualized CT features, and SUV_max_ were analyzed to identify independent predictors of STAS. A prediction model was built using the independent predictors and validated using the three chronologically separated cohorts. Model performance was assessed using ROC curves and calculations of AUC.

**Results:**

The differences in age (*P* = 0.009), lesion density subtype (*P* < 0.001), spiculation sign (*P* < 0.001), bronchus truncation sign (*P* = 0.001), and SUV_max_ (*P* < 0.001) between the positive and negative groups were statistically significant. Age ≥ 56 years [*OR*(95%*CI*):3.310(1.150–9.530), *P* = 0.027], lesion density subtype (*P* = 0.004) and SUV_max_ ≥ 2.5 g/ml [*OR*(95%*CI*):3.268(1.021–1.356), *P* = 0.005] were the independent factors predicting STAS. Logistic regression was used to build the A-D-S (Age-Density-SUV_max_) prediction model, and the AUCs were 0.808, 0.786 and 0.806 in the training, validation, and test cohorts, respectively.

**Conclusions:**

STAS was more likely to occur in older patients, in solid lesions and higher SUV_max_ in stage I LAC. The PET/CT-based A-D-S prediction model is easy to use and has a high level of reliability in diagnosing.

## Introduction

Lung cancer is a deadly disease with the second highest incidence and the highest mortality among all cancers worldwide, and lung adenocarcinoma (LAC) is the most common histological subtype of lung cancer [1, 2]. Spread through air spaces (STAS) which is defined as “micropapillary clusters, solid nests, or single tumor cells within airspaces beyond the edge of the main tumor”, as a new pathologic feature of tumor invasion, was formally proposed by the World Health Organization (WHO) classification of lung cancer in 2015 [3, 4]. In accordance with most literature reports, the incidence of STAS is approximately 15–40% [5]. Poor prognosis in lung cancer can be attributed to this important risk factor [6–8]. For stage I-III LAC, patients without STAS have significantly better recurrence-free survival (RFS) and overall survival (OS) compared to those with STAS [9]. This observation suggests that STAS-positive patients might warrant closer clinical follow-up. A retrospective study found that, for patients with STAS-positive stage I LAC, the risk of 5-year recurrence-free probability (RFP) after sublobar resection was significantly higher than that after lobectomy (48% vs. 66%; *P* = 0.010) [10]. This means that it is more necessary for such STAS-positive patients to undergo lobectomy to reduce the possibility of recurrence. Therefore, it is important to accurately predict STAS before operation in order to decrease the risk of relapse in patients with stage I LAC.

Several CT signs have been demonstrated to predict STAS, including density, CT long diameter, spiculation and so on [6, 11–16]. 2-deoxy-2-[^18^F]fluoro-D-glucose (2-[^18^F] FDG) positron emission tomography–computed tomography (PET/CT) imaging can simultaneously reflect the morphological and metabolic characteristics of the lesion, which play important roles in the diagnosis of lung cancer and identification of distant metastases [17]. The maximum standardized uptake value (SUV_max_) has also been shown to be of great importance in predicting STAS before surgery [6, 12].

Our objective was to create a user-friendly model that can predict the STAS status of patients with stage I LAC who have undergone preoperative 2-[^18^F] FDG PET/CT examination. The model includes only clinical and imaging data that can be directly accessed by thoracic surgeons, which is significant. The optimization of the surgical plan can be achieved by predicting the STAS status before surgery. By screening out high-risk patients who are more suitable for lobectomy, the likelihood of tumor recurrence can be minimized.

## Materials and methods

### Patients

This study complies with the principles of the Declaration of Helsinki and was approved by the Ethics Committee of the Affiliated Hospital of Qingdao University (Approval Number: QYFY WZLL 27218).

During January 2019 to December 2022, 466 patients with stage I LAC who received complete resection of the primary lung tumor and standard lymph node dissection at our hospital were retrospectively analyzed for data collection, including 161 males and 305 females (median age, 62 years; interquartile range, 55–67 years).

**Fig. 1:**
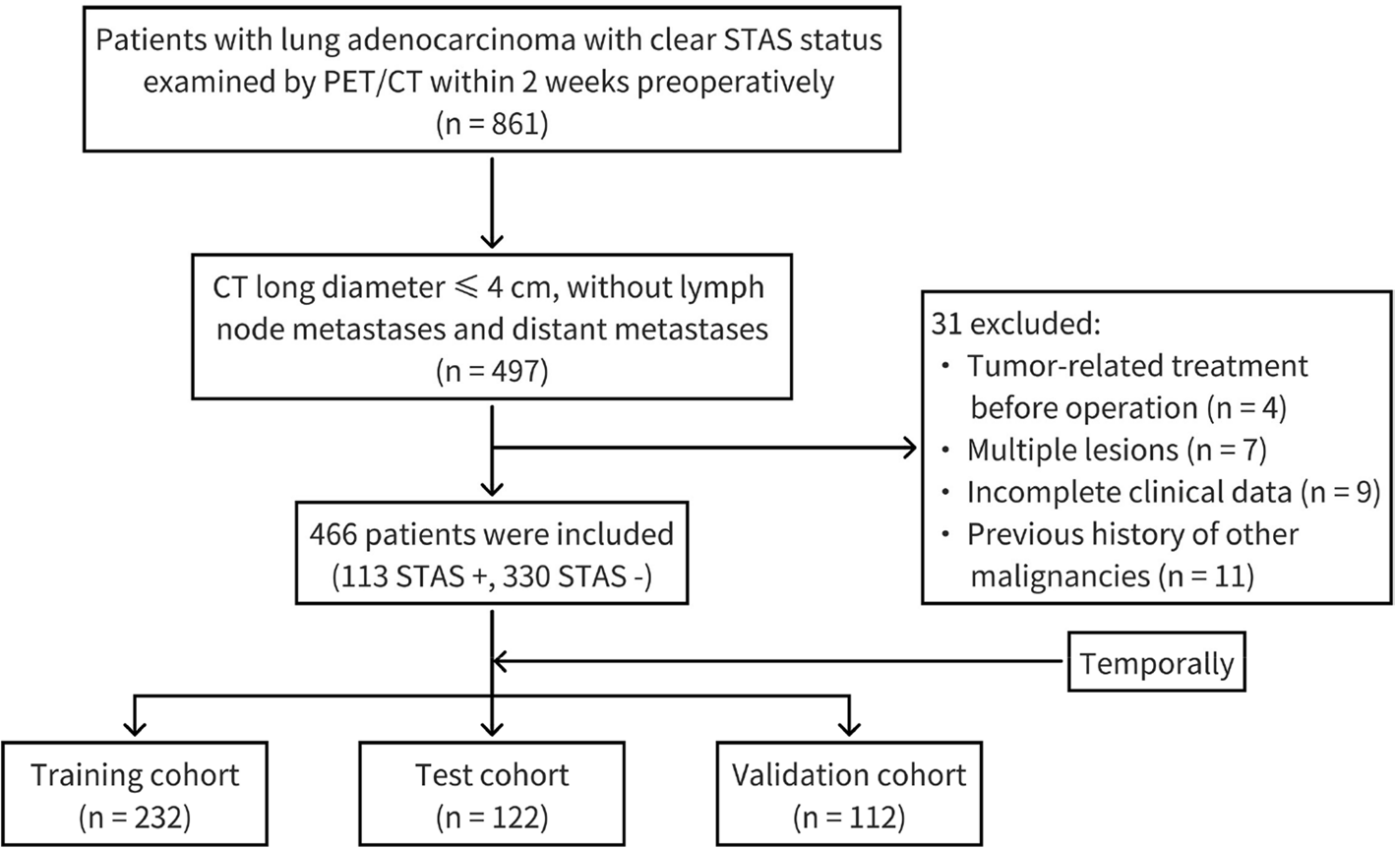
Flow chart of the inclusion and exclusion criteria. *STAS*, spread through air space

Inclusion criteria: The 2-[^18^F] FDG PET/CT was performed within 2 weeks preoperatively; the maximum diameter of lesions in CT images ≤4 cm; and the postoperative pathology confirmed the LAC without lymph node metastases and a clear STAS status. Patients received tumor-related treatment before operation (*n* = 4), patients with multiple lesions (*n* = 7), incomplete clinical data (*n* = 9), or previous history of other malignancies (*n* = 11) were excluded (Fig. 1).

According to the postoperative pathological results, all patients were classified as either STAS-positive or STAS-negative. The patients were grouped into a training cohort (January 2019 to December 2020; 232 cases), a validation cohort (January 2021 to December 2021; 122 cases), and a test cohort (January 2022 to December 2022; 112 cases) based on the date of surgery.

### Clinical data collection

Clinical information was obtained through an electronic medical record system, including age, sex, localization, and serum levels of carcinoembryonic antigen (CEA) within 3 weeks prior to surgery.

### Imaging method and image analysis

The 2-[^18^F] FDG radiotracer was prepared using a cyclotron (Sumitomo Heavy Industries, Ltd. Tokyo, Japan) automated synthesis module, with radiochemical purity > 95% and pH 4–8. Patients were injected with 5.5–6.6 MBq/kg (0.1 mCi/kg) of 2-[^18^F] FDG under the premise of fasting for at least 6 hours and plasma glucose of less than 11.1 mmol/L. One hour later, the bladder was emptied and then PET/CT images were collected. 2-[^18^F] FDG PET/CT was performed on a GE Discovery VCT PET/CT scanner, with the scanning range from the skull base to the mid-thigh level. A CT scan (scanning parameters: slice thickness 5 mm, tube current 110 mA, tube voltage 120 kV, frame rotation speed 0.7 r/s, bed speed 29.46 cm/s, matrix 512 × 512) was done first, followed by a PET scan (scanning parameters: matrix 128 × 128, 8–9 bed positions, 1.5–3.0 min/bed position). CT and PET images, which were reconstructed with decay correction and ordered subset expectation maximization (OSEM) algorithms were fused and reviewed on a Xeleris workstation. In addition, all patients involved underwent deep-inspiration breath-hold chest thin-slice CT scan (scanning parameters: layer thickness 1.25 mm, matrix 512 × 512); preset lung window (window width 1200 Hounsfield units (HU), window level − 700 HU).

The morphologic characteristics on CT and SUV_max_ of the lesions were independently analyzed by two Nuclear Medicine physicians who had more than 5 years of experience in the interpretation of PET/CT images. In cases where there is a disagreement between them, a physician with over 10 years of experience would join them, and they eventually came to a consensus through discussion. They were blinded to pathological details when reading the images. The lesion density was classified into three subtypes: pure ground-glass, part-solid, and solid. Other morphologic characteristics included CT long diameter, lobulation, spiculation, satellite, air bronchogram, vessel convergence, and bronchial truncation.

### Adjudication of STAS status

Hematoxylin-eosin (HE) sections and immunohistochemical sections of all histological samples of lung tissue were observed under a light microscope. When micropapillary clusters, solid nests, or single tumor cells are observed beyond the edge of the tumor into air spaces in the surrounding lung parenchyma, STAS is diagnosed after being checked correctly by two thoracic pathologists [3].

### Statistical analysis

IBM SPSS v26.0 and GraphPad Prism v9.5.1 were used for statistical processing and graphing. We express quantitative variables as mean ± standard deviation (^−^X ± SD) or median (quartile) [M (Q1, Q3)] and qualitative variables as frequencies (percentages). The categorical data were analyzed using χ^2^ test. Because all measurement data did not meet the normal distribution, Mann–Whitney U test was used for analysis. Independent predictors of STAS were selected by univariate and multivariate logistic regression analyses performed on the clinical data, CT features, and SUV_max_ in the training cohort. Subsequently, a prediction model was built using the independent predictors by logistic regression and validated through the three chronologically separated cohorts. Model performance was assessed using receiver operating characteristic (ROC) curves and calculations of area under the curves (AUC). The Hosmer-Lemeshow test was employed to measure the goodness-of-fit. All mentioned *P*-values were two-tailed and a *Ρ* < 0.05 was considered statistically significant.

## Results

### Patients’ data

In the training cohort, STAS was positive in 47 (20.3%) of the 232 subjects. In the validation cohort, STAS was positive in 33 (27.0%) of the 122 subjects. In the test cohort, STAS was positive in 33 (29.5%) of the 112 subjects. The distribution of pathological characteristics among the three cohorts of patients did not differ statistically (*χ*^2^ = 4.19, *P* > 0.05). The univariate analysis results showed that the differences of age (*z* = − 2.61, *P* = 0.009), lesion density subtype (*χ*^2^ = 38.60, *P* < 0.001), spiculation sign (*χ*^2^ = 14.53, *P* < 0.001), bronchus truncation sign (*χ*^2^ = 10.41, *P* = 0.001), and SUV_max_ (z = − 5.58, *P* < 0.001) between positive and negative groups were statistically significant (Table 1). According to the maximum value of Youden’s index of the ROC curve, the age of 56 years and SUV_max_ of 2.5 g/ml were identified as the optimal cut-off values.

**Table 1:** Clinical factors, CT features, and SUV_max_ of the patients in the training cohort

Variables	STAS status		Sig.	*P*-value
	Positive (*n* = 47)	Negative (*n* = 185)		
Sex, male [n, (%)]	19 (40.4%)	55 (29.7%)	1.974	0.160
Age [year, *M* (*Q*_1_*, Q*_3_)]	64 (58,69)	62 (54,66)	−2.611	0.009^*^
Age ≥ 56 years	42 (89.4%)	132 (71.4%)	6.484	0.013^*^
CEA [ng/ml, *M* (*Q*_1_, *Q*_3_)]	2.20 (1.45, 3.07)	1.98 (1.17, 3.05)	−0.903	0.368
CEA > 3.4 ng/ml	9 (19.1%)	35 (41.2%)	0.001	0.971
Localization—lung (left)	20 (42.6%)	72 (38.9%)	0.207	0.649
CT long diameter [mm, *M* (*Q*_1_, *Q*_3_)]	24.00 (17.70, 30.60)	21.20 (15.50, 28.75)	−1.396	0.163
Lesion density subtype			38.601	< 0.001^**^
pure ground-glass	3 (6.4%)	52 (28.1%)		
part-solid	12 (25.5%)	93 (50.3%)		
solid	32 (68.1%)	40 (21.6%)		
Lobulation	43 (91.5%)	155 (83.8%)	1.779	0.182
Spiculation	28 (59.6%)	55 (29.7%)	14.529	< 0.001^**^
Satellite	2 (4.3%)	3 (0.02%)	0.300	0.584
Air bronchogram	24 (51.1%)	86 (46.5%)	0.315	0.575
Vessel convergence	30 (63.8%)	111 (60.0%)	0.231	0.631
Bronchial truncation	13 (27.7%)	18 (9.7%)	10.408	0.001^*^
SUV_max_ [g/ml, *M* (*Q*_1_, *Q*_3_)]	3.20 (2.04, 7.82)	1.56 (0.99, 2.36)	−5.579	< 0.001^**^
SUV_max_ ≥ 2.5 g/ml	32 (68.1%)	43 (23.2%)	34.448	< 0.001^**^

*STAS* tumor spread through air spaces, *SUV*_*max*_ the maximum standardized uptake value

^*^Statistically significant, *P* < 0.05; ^**^Statistically significant, *P* < 0.001

**Fig. 2:**
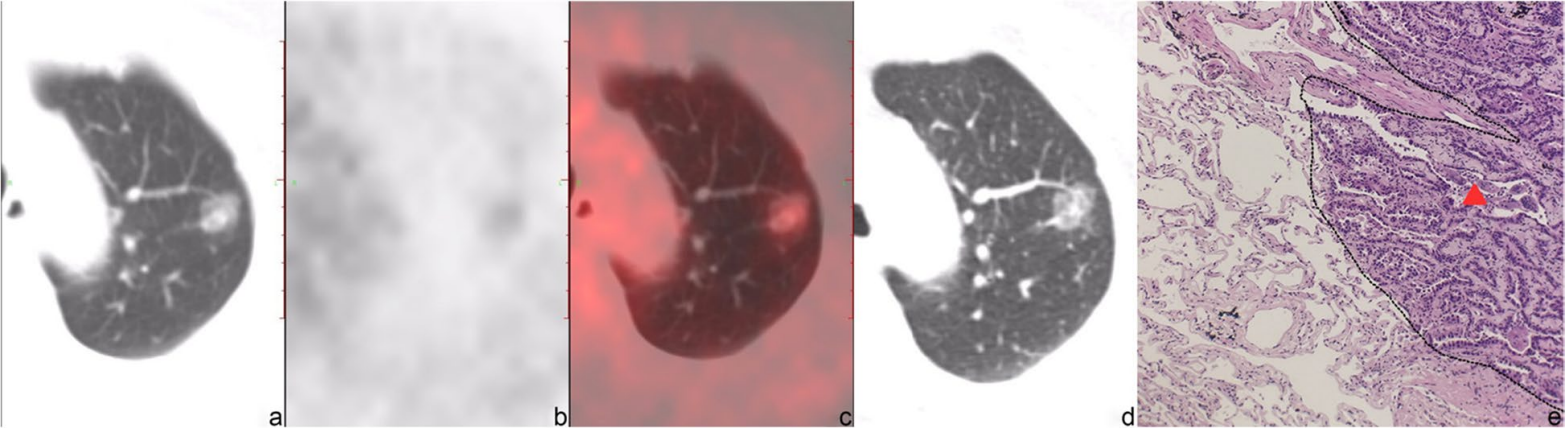
56-year-old female with invasive adenocarcinoma in the left upper lobe, STAS (−). On axial CT in the lung window (**a**) and axial chest thin-slice CT (**d**), a pure ground-glass lesion which is about 1.5 × 2.0 cm can be observed, lobulation sign and vessel convergence can be seen. Axial PET (**b**) and axial fused PET/CT (**c**) show the SUV_max_ of the lesion is 1.4 g/ml. Photomicrograph (hematoxylin-eosin stain, magnification × 400) (**e**) shows no tumor tissue in the alveolar cavity outside the edge (dashed line) of the tumor (triangle)

**Fig. 3:**
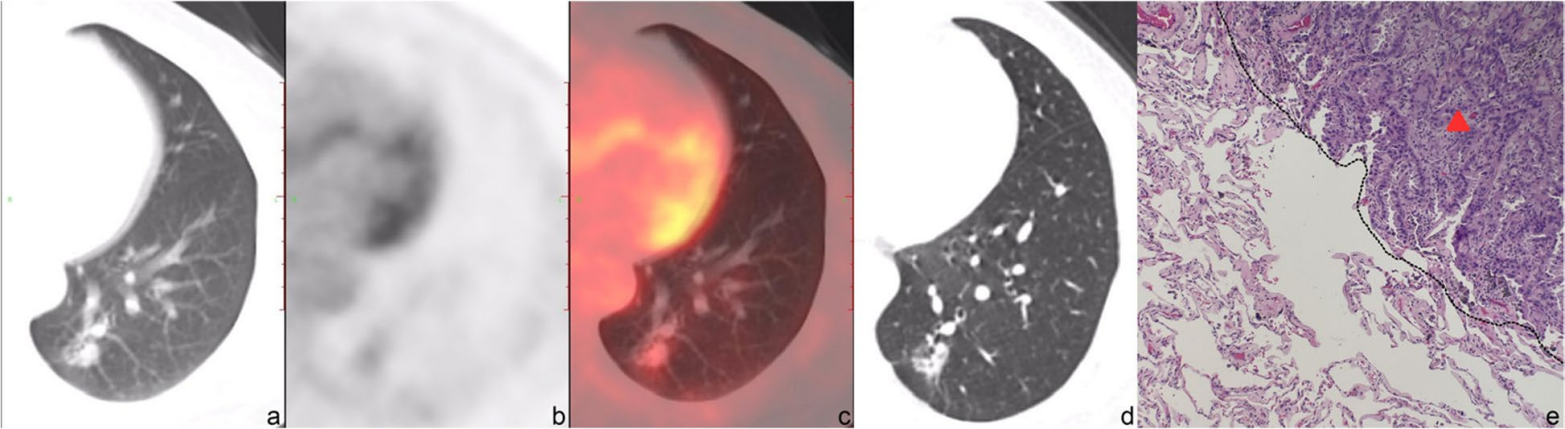
46-year-old female with invasive adenocarcinoma in the left lower lobe, STAS (−). On axial CT in the lung window (**a**) and axial chest thin-slice CT (**d**), a part-solid lesion which is about 1.1 × 1.6 cm can be observed. Axial PET (**b**) and axial fused PET/CT (**c**) show the SUV_max_ of the lesion is 1.9 g/ml. Photomicrograph (hematoxylin-eosin stain, magnification × 400) (**e**) shows no tumor tissue in the alveolar cavity outside the edge (dashed line) of the tumor (triangle)

**Fig. 4:**
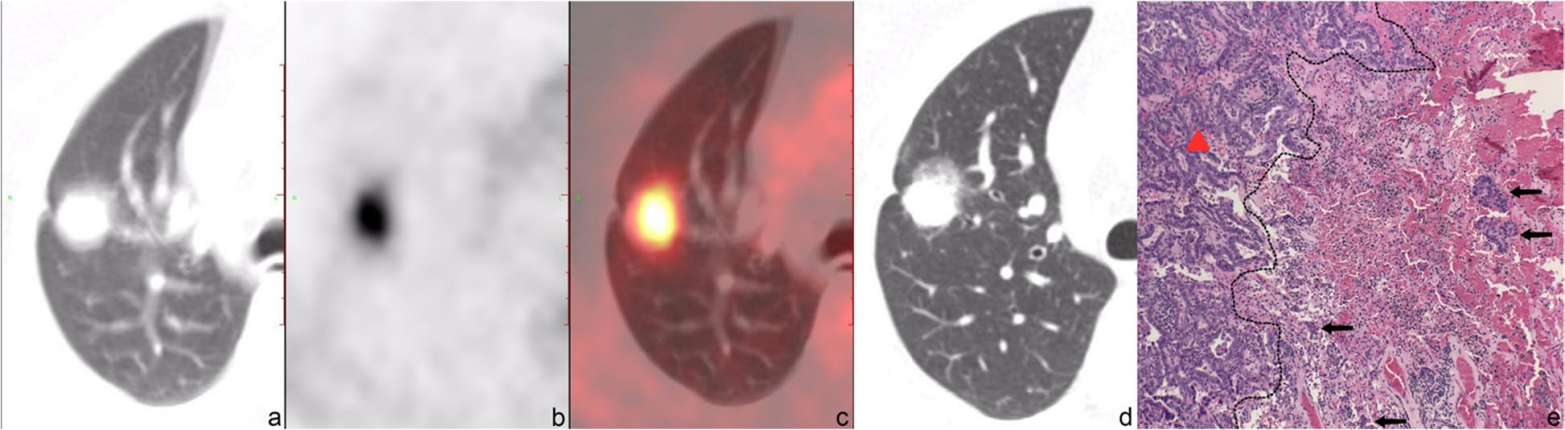
61-year-old female with invasive adenocarcinoma in the right upper lobe, STAS (+). Axial CT in the lung window (**a**) and axial chest thin-slice CT (**d**) show that the part-solid nodule is about 2.7 × 2.0 cm with lobulation, spiculation, pleural indentation and vessel convergence. Axial PET (**b**) and axial fused PET/CT (**c**) show the SUV_max_ of the lesion is 6.3 g/ml. In photomicrograph (hematoxylin-eosin stain, magnification × 400) (**e**), tumor tissues (black arrow) are observed in the alveolar spaces beyond the edge (dashed line) of the main tumor (triangle)

**Fig. 5:**
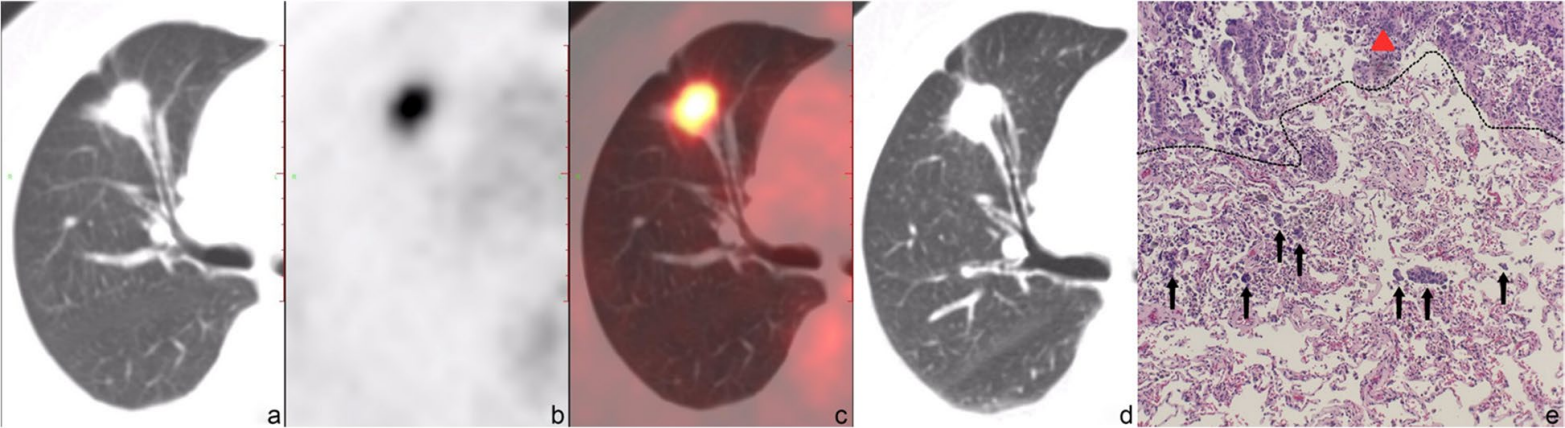
70-year-old female with invasive adenocarcinoma in the right upper lobe, STAS (+). Axial CT in the lung window (**a**) and axial chest thin-slice CT (**d**) show that the solid nodule is about 1.5 × 2.0 cm with lobulation, spiculation, vessel convergence and bronchial truncation. Axial PET (**b**) and axial fused PET/CT (**c**) show the SUV_max_ of the lesion is 7.2 g/ml. In photomicrograph (hematoxylin-eosin stain, magnification × 400) (**e**), tumor tissues (black arrow) are observed within air spaces in the surrounding lung parenchyma adjacent to the boundary (dashed line) of the bulk of the tumor (triangle). *STAS*, tumor spread through air spaces; *SUV*_*max*_, the maximum standardized uptake value

Typical PET/CT images and histopathological images for the two groups are displayed in Figs. 2, 3, 4 and 5. (Notes: (a) Axial CT images in the lung window; (b) Axial PET images; (c) Axial fused PET/CT images; (d) Axial chest thin-slice CT scan images in the lung window; (e) pathological images).

### Logistic regression analysis

The above statistically significant (*P* < 0.05) parameters were incorporated into univariate and multivariate logistic regression analysis. The results showed that age ≥ 56 years [*OR* (95%*CI*): 3.310 (1.150–9.530), *P* = 0.027], lesion density subtype (*P* = 0.004) and SUV_max_ ≥ 2.5 g/ml [*OR* (95%*CI*): 3.268 (1.021–1.356), *P* = 0.005] were the independent factors predicting STAS (Table 2).

**Table 2:** Univariate and multivariate logistic regression analysis of the independent association between risk factors and STAS

Risk factors	Univariate logistic regression			Multivariate logistic regression		
	*OR*	95%*CI*	*P*-value	*OR*	95%*CI*	*P*-value
Age ≥ 56 years	3.373	1.265–8.991	0.015^*^	3.310	1.150–9.530	0.027^*^
Lesion density subtype^*a*^			< 0.001^**^			0.004^*^
part-solid	2.237	0.604–8.288	0.228	1.165	0.282–4.813	0.833
solid	13.867	3.960–48.553	< 0.001^**^	3.268	1.416–7.539	0.043^*^
Spiculation	3.483	1.796–6.756	< 0.001^**^	1.452	0.642–3.286	0.371
Bronchial truncation	3.547	1.589–7.919	0.002^*^	1.485	0.574–3.841	0.415
SUV_max_ ≥ 2.5 g/ml	7.045	3.492–14.212	< 0.001^**^	3.268	1.416–7.539	0.005^*^

*STAS* tumor spread through air spaces, *OR* odds ratio, *SUV*_*max*_ the maximum standardized uptake value

^*^Statistically significant, *P* < 0.05; ^**^Statistically significant, *P* < 0.001; ^*a*^The pure ground-glass group was considered the reference

### Establishment and verification of the prediction model

Ultimately, the above three independent predictors were chosen to establish the A-D-S (Age-Density-SUV_max_) logistic regression risk prediction model: *P* = 1 / (1 + e^-x^), where e = 2.718…is the natural constant, x = − 3.871 + A + D + S. Among them, “A” assumes 1.243 when the age ≥ 56 years, otherwise it assumes 0; “D” is assigned to 0, 0.321, or 1.829 when the lesion density subtype is pure ground-glass, part-solid or solid, respectively; “S” takes 1.249 when SUVmax ≥2.5 g/ml, otherwise it takes 0. The model has a good fit (Hosmer–Lemeshow test: *P* = 0.959).

**Fig. 6:**
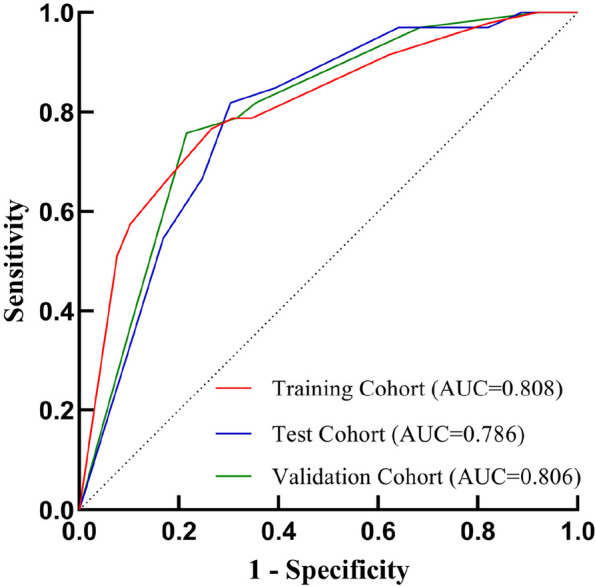
The ROC curves evaluating the predictive efficiency of the A-D-S risk prediction model. *ROC*, receiver operating characteristic; *AUC*, area under the curve; *A-D-S*, Age-Density-SUV_max_

In the training, validation and test cohorts, the AUCs of the A-D-S risk prediction model were 0.808 (95%*CI*: 0.738–0.880), 0.786 (95%*CI*: 0.700–0.872) and 0.806 (95%*CI*: 0.720–0.892), respectively; sensitivity was 0.766, 0.818, and 0.788, respectively; and specificity was 0.735, 0.697, and 0.684, respectively (Fig. 6).

## Discussion

The differentiation of benign and malignant pulmonary nodules and the risk stratification of lung cancer have always been the focus of clinical research, as an adverse prognostic factor for lung cancer, STAS has attracted widespread attention from clinicians, radiologists, and pathologists recently. Sublobar resection is one of the primary modes of treatment for stage I LAC [18–20]. However, according to a study, patients with STAS are at a greater risk of recurrence after sublobar resection [21]. Therefore, the STAS status of patients with stage I LAC affects the choice of surgical approach. The likelihood of recurrence can be reduced by judging the STAS status of the tumor preoperatively and performing lobectomy in patients with suspected positivity when conditions permit. Perhaps due to the limited scope of materials and other reasons, presurgical bronchial cytology is not sufficient to accurately predict tumor STAS [22], and the diagnostic efficacy of intraoperative frozen pathology is also controversial [21, 23, 24]. By using a simple and reliable method to predict the STAS status of lung cancer, patients could be stratified effectively, and surgical plans could be developed appropriately, which could potentially improve the prognosis of patients. This makes research on predicting STAS based on preoperative imaging a hot topic.

In this study, the age of STAS-positive patients was slightly older than that of STAS-negative patients, which was the same as the result reported by Chae et al. [25]. This may be related to the natural history of the tumor. It usually takes several years for carcinoma in situ to progress to microinvasive adenocarcinoma and then to invasive adenocarcinoma. During this process, as the tumor invasiveness increases, the cancer cells are more likely to dissociate into the airway outside the main body of the tumor and develop STAS. Jiang et al. [26] concluded that STAS-positive patients were younger, which may be related to the lack of staging screening when the study included cases. In short, the correlation between age and STAS needs to be further studied.

A quantitative study showed that every time the consolidation percentage on CT increased by 1%, the risk of STAS increased more than 3-fold in early non-small cell lung cancer [12]. We arrived at a similar conclusion: for stage I lung adenocarcinoma whose lesion density subtype was pure ground glass, part-solid and solid, the incidence of STAS increased gradually. The conclusion is also consistent with that reported by Kim et al. [11]. Moreover, this classification method avoids the errors caused by manual measurement when calculating the consolidation percentage. In addition, lung cancer with pure ground glass density on CT was considered to be free of STAS in most previous studies. However, in this study, STAS also appeared in these lesions with a small probability (4/103, 3.88%), which was consistent with the findings of previous studies [16, 27].

SUV value is the most commonly used semi-quantitative index of 2-[^18^F] FDG PET/CT, which can reflect the activity of glucose metabolism in tumor tissue and is closely related to the degree of risk and biological invasiveness of tumor [28]. In this study, the occurrence probability of STAS was positively correlated with SUV_max_, which could be explained by the greater metabolic activity and aggressiveness of STAS-positive LAC. Furthermore, we found that SUV_max_ ≥ 2.5 g/ml was the optimal cut-off value to predict STAS, which is coincidentally consistent with previous research results [6].

In recent years, several models have been proposed to predict STAS and have achieved good prediction performance. For example, a model established by Liao et al. [29] based on radiomics to predict STAS of clinical stage I LAC achieved an AUC of 0.871 (95%*CI*: 0.820–0.922) and 0.869 (95%*CI*: 0.776–0.961) in the validation and test cohorts, respectively. It can be seen that the diagnostic performance of this model is indeed higher than that of the A-D-S model. However, the advantage of the A-D-S model is that it is easier to use and more timesaving. Li et al. [14] developed a CT-based logistic regression prediction model that achieved AUCs of 0.801 (95%*CI*: 0.709–0.892) and 0.692 (95%*CI*: 0.518–0.866) in the validation and external test cohorts, respectively. The diagnostic efficiency of this model is similar to that of A-D-S, but they did not stage the tumor when they included the data, which might lead to an increase in confounding factors, further resulting in exaggeration or underestimation of the prediction performance. In our study, we specifically targeted clinical stage I LAC, and the three parameters included in the model could be obtained directly from medical records and PET/CT reports and images without complex post-processing. Therefore, as a user-friendly model, compared with the above two studies, the A-D-S prediction model has better clinical practicality.

The deficiencies of this study are as follows. First, the CT imaging features included in the study are subjective. To guarantee the repeatability of these factors, we employed 2–3 senior doctors to read the images. Second, because this was a single-center study, we used temporal validation to verify the effectiveness of the model. Although this method is better than internal validation, it is evident that there are numerous similarities among the three patient cohorts and among the clinical and laboratory techniques employed in their evaluation. Therefore, the evaluation of the generalization ability of this verification method is not as good as that of external verification [30]. Third, the sample size was relatively small. Follow-up large-scale multicenter prospective studies are needed to confirm the conclusions of this study, to better provide a clinic basis.

## Conclusion

The STAS status of stage I lung adenocarcinoma is related to multiple PET/CT imaging features. Age, lesion density subtype, and SUV_max_ are independent predictors of STAS in stage I LAC. This study included the above three factors to establish a STAS risk prediction model. The model has good prediction performance. More importantly, it can be conveniently used in the clinic to evaluate the STAS status of stage I lung adenocarcinoma before surgery. It can help thoracic surgeons optimize surgical procedures with a view to improving patient prognosis.

## Data Availability

All the data generated and analyzed during this study are included in our manuscript. The data supporting the findings of this study are available from the corresponding author upon reasonable request.
